# HER2/neu-Based Peptide Vaccination-Pulsed with B-Cell Epitope Induced Efficient Prophylactic and Therapeutic Antitumor Activities in TUBO Breast Cancer Mice Model

**DOI:** 10.3390/cancers13194958

**Published:** 2021-10-01

**Authors:** Muhammad Luqman Nordin, Abdin Shakirin Mohamad Norpi, Pei Yuen Ng, Khatijah Yusoff, Nadiah Abu, Kue Peng Lim, Fazren Azmi

**Affiliations:** 1 Centre for Drug Delivery Technology, Faculty of Pharmacy, Universiti Kebangsaan Malaysia (UKM) Kuala Lumpur Campus, Jalan Raja Muda Abdul Aziz, Kuala Lumpur 50300, Malaysia; luqman.n@umk.edu.my (M.L.N.); abdin22shah@gmail.com (A.S.M.N.); 2 Department of Clinical Studies, Faculty of Veterinary Medicine, Universiti Malaysia Kelantan (UMK), Pengkalan Chepa, Kota Bharu 16100, Kelantan, Malaysia; 3 Faculty of Pharmacy and Health Sciences, Royal College of Medicine Perak, Universiti Kuala Lumpur, No.3 Jalan Greentown, Ipoh 30450, Perak, Malaysia; 4 Drug and Herbal Research Centre, Faculty of Pharmacy, Universiti Kebangsaan Malaysia (UKM), Jalan Raja Muda Abdul Aziz, Kuala Lumpur 50300, Malaysia; pyng@ukm.edu.my; 5 UPM-MAKNA Cancer Research Laboratory, Institute of Bioscience, Universiti Putra Malaysia (UPM), Serdang, Seri Kembangan 43400, Selangor, Malaysia; kyusoff@upm.edu.my; 6 UKM Medical Molecular Biology Institute (UMBI), UKM Medical Centre, Jalan Ya’acob Latiff, Bandar Tun Razak, Cheras, Kuala Lumpur 56000, Malaysia; nadiah.abu@ppukm.ukm.edu.my; 7 Cancer Immunology & Immunotherapy Unit, Cancer Research Malaysia, No. 1 Jalan SS12/1A, Subang Jaya 47500, Selangor, Malaysia; kuepeng.lim@cancerresearch.my

**Keywords:** multi-epitope, peptide vaccines, antitumor, HER2/neu, B-cell epitope, breast cancer

## Abstract

**Simple Summary:**

Recently, the role of vaccination has been expanded in the management of HER2/neu-positive breast cancer. Data from various clinical trials indicated that an effective breast cancer vaccine must be able to induce both humoral and cellular immune responses. One approach is the use of a multi-epitope peptide vaccine comprising B, T helper and CTL epitopes. In this study, we have successfully developed a vaccine construct based on a chimeric HER2/neu-derived antigenic peptide possessing B- and T-cell epitopes conjugated to the KLH protein as a source of T-helper epitopes. A similar vaccine construct, but without the presence of a B-cell peptide epitope, was also prepared to examine the role of the humoral immune response in antitumor immunity. Multi-epitope peptide vaccine with additional B-cell epitope was found to be superior in inhibiting the tumor growth and prolongation of survival in tumor mice model compared to vaccine construct without the presence of B-cell epitope.

**Abstract:**

Breast cancer is the most common invasive cancer diagnosed among women. A cancer vaccine has been recognized as a form of immunotherapy with a prominent position in the prevention and treatment of breast cancer. The majority of current breast cancer vaccination strategies aim to stimulate antitumor T-cell responses of the HER2/neu oncogene, which is abnormally expressed in breast cancer cells. However, the role of the B-cell humoral response is often underappreciated in the cancer vaccine design. We have advanced this idea by elucidating the role of B-cells in cancer vaccination by designing a chimeric antigenic peptide possessing both cytotoxic T lymphocytes (GP2) and B-cell (P4) peptide epitopes derived from HER2/neu. The chimeric peptide (GP2–P4) was further conjugated to a carrier protein (KLH), forming a KLH–GP2–P4 conjugate. The immunogenicity of KLH–GP2–P4 was compared with KLH–GP2 (lacking the B-cell epitope) in BALB/c mice. Mice immunized with KLH–GP2–P4 elicited more potent antigen-specific neutralizing antibodies against syngeneic TUBO cells (cancer cell line overexpressing HER2/neu) that was governed by a balanced Th1/Th2 polarization in comparison to KLH–GP2. Subsequently, these immune responses led to greater inhibition of tumor growth and longer survival in TUBO tumor-bearing mice in both prophylactic and therapeutic challenge experiments. Overall, our data demonstrated that the B-cell epitope has a profound effect in orchestrating an efficacious antitumor immunity. Thus, a multi-epitope peptide vaccine encompassing cytotoxic T-lymphocytes, T-helper and B-cell epitopes represents a promising strategy in developing cancer vaccines with a preventive and therapeutic modality for the effective management of breast cancer.

## 1. Introduction

Breast cancer remains the most prevalent type of cancer in women. Globally, there were approximately 2.3 million new cases of women diagnosed with breast cancer in 2020, and it was accountable for 685,000 casualties [1]. Drug resistance, epigenetic mutations, unnecessary side effects and high propensity for relapse continue to be significant challenges in conventional breast cancer therapy [2,3]. Comprehensive profiling of tumor immunogenicity has paved the way for the development of immunotherapies against cancer [4]. Cancer vaccines, which are an active form of immunotherapy, have emerged as one of the most effective and safe strategies for managing cancer. They work by stimulating an immune response that targets specific molecules, commonly known as tumor-associated antigens (TAAs), that are expressed at elevated levels on cancer cells [5].

Human epidermal growth factor receptor-2/neu (HER2/neu) is one of the most well-studied TAAs in the development of breast cancer vaccines [6]. HER2/neu protein is overexpressed in approximately 20–30% of primary breast cancers and is associated with poor prognosis outcomes [7,8]. Peptide-based vaccines confer several advantages over conventional vaccines (e.g., attenuated forms of the whole pathogen and recombinant protein-based vaccine), such as being capable of eliciting a highly specific immune response, thus eliminating unnecessary autoimmune reactions [9], as well as simplicity in the production and a good safety profile [10,11]. Even in DNA-based vaccines, it is still encoded for the production of an antigenic protein that may contain unnecessary epitopes that may induce unfavorable reactogenic responses. This rationale led to an interest in focusing on a limited set of epitopes that only represent the minimal immunogenic determinant of a protein antigen. A highly immunogenic peptide, GP2 (654–662: IISAVVGIL), which is derived from the transmembrane domain of the HER2 protein, has been identified to induce a cytotoxic T-lymphocyte (CTL) response [12,13]. The CTL-mediated immune response is considered essential for antitumor activity. However, clinical trials data demonstrated that the use of CTL peptide alone in cancer vaccines was insufficient for the complete eradication of cancer cells [14,15,16]. These poor clinical outcomes have necessitated the need for a new strategy in designing a more efficacious peptide-based cancer vaccine. Generally, peptides only represent a minimal component of a protein; thus, they are poorly immunogenic on their own [17]. One of the well-established strategies to enhance the peptide’s immunogenicity is to covalently link them to a carrier protein, such as keyhole limpet hemocyanin (KLH). KLH is a highly immunogenic protein enriched with T helper (Th) epitopes and has been successfully used as immunostimulants in various human clinical research settings, including studies on infectious, immunotoxicological and autoimmune diseases [18,19]. The importance of Th cells in stimulating antitumor immunity via enhancing and priming effector T-cells, particularly CTL, is well documented [20].

Recently, it has been appreciated that B-cells play a significant role in tumor immunity. B-cells have diverse roles in tumor microenvironment, including the ability to stimulate T-cells and innate immunity in addition to elicit antibody secretion [21,22]. For these reasons, the inclusion of B-cell epitopes may provide additional benefits in enhancing the vaccine-induced CTL antitumor immune responses. Various B-cell epitopes from the HER2/neu receptor’s extracellular domain have been identified and are being tested for vaccine development [23,24]. Among them, an immunodominant B-cell peptide epitope denoted as P4 (aa 378–398: PESFDGDPASNTAPLQPEQLQ) was validated in preclinical studies and has demonstrated potent ability to induce HER-2/neu specific antibodies with antitumor activity [25].

**Figure 1:**
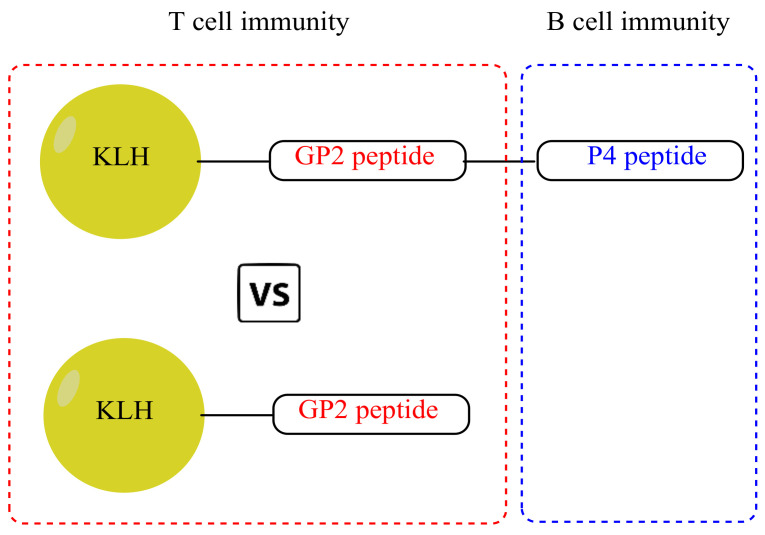
Schematic structure of the HER2/neu-based multi-epitope peptide vaccine candidates.

Building up from this knowledge, we hypothesized that a multi-epitope vaccine encompassing epitopes that can activate CTL, Th and B-cells could induce a potent immune response against the targeted cancer cells. In this study, we have constructed HER2/neu-based multi-epitope peptide vaccine for breast cancer consisting of GP2 (CTL epitope) and P4 (B-cell epitope) peptide that was covalently linked to KLH, as a source of Th cells stimulation and abbreviated as KLH–GP2–P4 (Figure 1). In order to elucidate the role of B-cell epitope in inducing significant antitumor immunity, KLH conjugated to GP2 alone (KLH–GP2) was also included as part of the accessed vaccine candidates. The constructed HER2/neu peptide-based vaccine candidates were administered in both prophylactic and therapeutic vaccination settings in the TUBO tumor model of BALB/c mice, which overexpresses the HER2/neu oncogene. Their tumor inhibition activity was demonstrated by examining the tumor volumes and mice survivability. Cytokine profiling, antibody titers determination and tumor neutralization assay were performed to evaluate the immunogenicity of the constructed vaccine candidates in inducing HER2/neu-specific humoral and cellular immune responses. The safety and toxicity of each vaccine were assessed based on the results of physical examinations, differences in body weights, injection site reactogenicity, serum biochemistry profile and histopathological evaluations on important organs. Data emanating from this study will provide a new paradigm in designing an effective peptide-based cancer vaccination strategy.

## 2. Materials and Methods

### 2.1. Materials

Dulbecco’s Modified Eagle Medium (DMEM) with L-glutamine, fetal bovine serum (FBS), antibiotic-antimycotic (10,000 units/mL of penicillin, 10,000 μg/mL of streptomycin and 25 μg/mL amphotericin B), 0.25% trypsin-EDTA, phosphate buffer saline (PBS) and RIPA lysis buffer 1× were purchased from Thermo Fisher Scientific (Waltham, MA, USA). Microculture tetrazolium (MTT) powder and PrimePlex Mouse 4-plex Proteomics Assay Kit (MILLIPLEX^®^, Merck Millipore, Burlington, MA, USA) were purchased from Prima Nexus Sdn Bhd (Wilayah Persekutuan Kuala Lumpur, Malaysia). The Duoset ancillary reagent kit 2 was purchased from BD Biosciences (Franklin Lakes, NJ, USA). Romanowsky stain, methylene blue, ethanol, methanol, and dimethyl sulfoxide were purchased from Sigma-Aldrich (Saint Louis, MO, USA). HER-2 antibody was purchased from Novus Biologicals (Littleton, CO, USA). Xylazine-100 and Ketamine hydrochloride were obtained from the Laboratory Animal Research Unit, Faculty of Medicine, UKM, Kuala Lumpur. GP2 (Ac- CSGSGIISAVVGIL) and GP2–P4 (Ac CSGSGIISAVVGILSGSGPESFDGDPASNTAPLQPEQLQ) with molecular weight 1317.57 Da and 3829.19 Da, respectively, were purchased from GL Biochem (Shanghai, China).

### 2.2. Cell Culture

Human embryonic kidney 293 (HEK) cells were obtained from American Type Culture Collection (ATCC), USA. TUBO (overexpressing the HER2/neu protein), a cloned cell line from a spontaneous mammary tumor in a BALB-neuT, was generously provided by Prof. Dr. Pier-Luigi Lollini (Department of Clinical and Biological Sciences, University of Turin, Orbassano, Italy). TUBO cells were grown in DMEM media with L-glutamine, supplemented with 10% fetal bovine serum (FBS) and 1% antibiotic-antimycotic (10,000 units/mL of penicillin, 10,000 μg/mL of streptomycin and 25 μg/mL amphotericin B) as a complete growth medium (CGM) at 37 °C with 5% CO_2_ in the incubator.

### 2.3. Protein–Peptide Conjugates

GP2 (Ac- CSGSGIISAVVGIL) and GP2–P4 (Ac CSGSGIISAVVGILSGSGPESFDGDPASNTAPLQPEQLQ) with purity > 95% were synthesized and characterized by high-performance liquid chromatography (HPLC) and mass spectrometry analysis by GL Biochem (China) Ltd. All peptides contained the linker sequence Ac-CSGSG. In addition, the GP2–P4 peptide also contained additional SGSG between the sequence of GP2 and P4. Both GP2 and GP2–P4 were conjugated to KLH via mercapto Maleimide chemistry, in which the cysteine residue of the synthetic peptides was coupled to Maleimide-modified KLH. The conjugation between KLH and peptides was confirmed via sulfhydryl group test analysis. The conjugation procedure was performed by the same company. Lyophilized KLH–GP2–P4 and KLH–GP2 were stored at −20 °C until further use.

### 2.4. Experimental Animal

Six-week-old female BALB/c mice weighing 20–22 g were obtained from the Universiti Kebangsaan Malaysia (UKM) Laboratory Animal Research Unit (Malaysia). The mice were acclimatized for seven days before the experiment began. They were housed in cages (n = 6 per group) and fed ad libitum with pelleted commercial diets pellets and water. The cages were cleaned with 70% alcohol, and the bedding was changed on regular basis. Housing conditions were maintained at 26 °C ± 2 °C, with 12 h daylight and 12 h dark cycle lighting controls. All animal experiments were conducted in accordance with the protocols approved by UKM Animal Ethics Committee (UKMAEC) (approval number: FF/2020/FAZREN/14-MAY/1103-MAY-2020-MAY-2023).

### 2.5. Mice Immunization for Immunogenicity Evaluations

**Figure 2:**
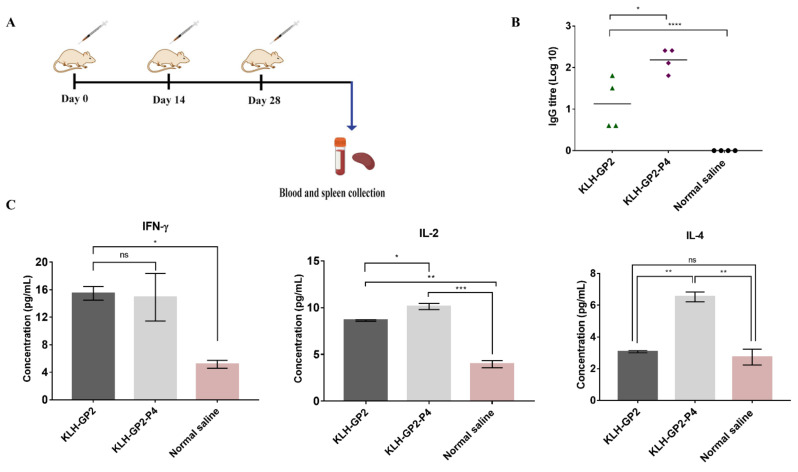
(**A**) The schematic protocol of BALB/c mice immunization with peptide-based vaccine candidates. Sera and spleen of immunized mice were collected two weeks after the final vaccination. (**B**) HER2/neu-specific IgG antibody titers in serum. Each point in the figure represents an individual mouse (*n* = 4), and the mean antibody titers are represented as a bar. (**C**) Level of splenocyte cytokines response prior to final immunization. The data represent mean concentrations ± SEM. Statistical analysis was performed using one-way ANOVA followed by Tukey’s multiple comparison test (ns, *p* > 0.05; * *p* < 0.05; ** *p* < 0.01; *** *p* < 0.001, and **** *p* < 0.0001).

The BALB/c mice were divided into three groups (*n* = 4 mice per group). Mice were immunized subcutaneously at the neck region with 30 µg of either KLH–GP2–P4 or KLH–GP2 in a total volume of 100 μL of normal saline three times at two-week intervals (Figure 2A). The negative control group received normal saline at a total volume of 100 μL. Two weeks after the final vaccination, mice were euthanized with ketamine (80–120 mg/kg)/xylazine (5–16 mg/kg), and blood samples from each mouse were collected via cardiac puncture. The blood samples were centrifuged to extract the sera. The collected sera were stored at −80 °C until further analysis. Selected vital organs: liver, kidney and heart were dissected out and fixed in 4% buffered formalin (*v*/*v*) for preservation until further used.

### 2.6. Reactogenicity and Safety Assessment of the Vaccine Candidates

The general safety assessment was carried out following the established protocol [26]. The BALB/c mice were closely monitored for any clinical abnormalities and toxicity symptoms throughout the immunization period. The injection site was observed at 1, 3, 24, 48 and 72 h after the first dose of immunization for any signs of inflammation, erythema, seroma, edema and necrosis. The weight of the mice was measured using analytical balance (Mettler Toledo MS204S) on a weekly basis. The percentage of body weight changes (%) was calculated according to the following formula:

$$\frac{\mathrm{Body}\;\mathrm{weight}\;\mathrm{of}\;\mathrm{each}\;\mathrm{week}\;\left(g\right)-\mathrm{Body}\;\mathrm{weight}\;\mathrm{of}\;\mathrm{the}\;\mathrm{initial}\;\mathrm{weight}\left(g\right)}{\mathrm{Initial}\;\mathrm{body}\;\mathrm{weight}\;\left(g\right)}\times 100\%$$

### 2.7. Serum Biochemical Analyses

Two hundred microliter of collected serum were subjected to biochemical analysis. Creatinine (CREA), aspartate aminotransferase (AST) and alanine aminotransferase (ALT) were the biochemical parameters analyzed in the serum. Biochemical analyses were performed using the IDEXX VetTest Chemistry analyzer.

### 2.8. Histopathological Examination

The liver, kidney and heart organs were dissected and isolated from the immunized mice. The collected organs (harvested and fixed in 10% buffered formalin) were sectioned into 0.5–1 cm thickness and placed into cassettes. The paraffin was added to form a block around the sample by using a processor machine. The tissues were sliced into 4 µm thick slices using a microtome and dried at 37 °C. Sectioned tissues were stained with hematoxylin and eosin (H & E). All tissue samples were examined for a histopathological lesion by light microscopy on an Olympus BX40-B microscope at a magnification of 40×.

### 2.9. Cytokines Quantification

A panel of cytokines (INF-γ, IL-2 and IL-4) were quantified using a PrimePlex Proteomics Assay Immunoassay (MILLIPLEX^®^, Merck Millipore, Burlington, MA, USA) following the manufacturer’s protocol. At the time of sacrifice, spleens (100 mg) from the immunized mice were immersed in 300 mL of 1× radioimmunoprecipitation assay buffer (RIPA buffer) and homogenized using a probe sonicator (Qsonica, Newtown, CT, USA) at 25 Hz for 1–3 min to obtain a single-cell suspension. Culture supernatants were isolated by centrifugation at (14,000× *g* for 10 min at 4 °C). The cytokines expression in the culture supernatants was quantified based on mean fluorescence intensities, and the curve fitting was compared to the master calibrator curves provided by the manufacturer (Luminex’s xMAP^®^ Technology, Austin, TX, United States). All samples were run in triplicate.

### 2.10. HER2/neu-Specific Antibody Titers Evaluation

Antibody (IgG) titers were determined using an enzyme-linked immunosorbent assay (ELISA) based on the protocol described elsewhere with slight modification [27]. Briefly, 10 µg/mL HER2/neu was coated onto ELISA plates (100 µL/well) in the presence of carbonate coating buffer and incubated at 27 °C for 24 h. The plates were washed twice with a washing buffer and rinsed to remove any residual after the overnight incubation. Then, 200 µL/well of blocking buffer (3% BSA in PBS with 0.05% Tween-20) was added to the ELISA plates and incubated at 37 °C for 1 h. Aliquots of murine sera (1:100) with reagent diluent were added to each well in the plate, followed by 1:2 dilutions down the plate. Then, 100 µL of secondary antibody (horseradish peroxidase-conjugated goat anti-mouse secondary antibody IgG) was diluted in 0.1% reagent diluent was added to each well in the plate and incubated for 2 h at room temperature. 100 µL mixture of color reagent A (stabilized hydrogen peroxide) and B (stabilized tetramethylbenzidine) (1:1) was added to each well and incubated for 20 min at 25 °C in the darkroom. Absorbance data were determined at a wavelength of 450 nm using an Infinite M200 PRO TECAN, (Tecan Trading AG, Männedorf, Switzerland) microplate reader. Antibody titers were defined as the lowest dilution that gave an absorbance of three standard deviations above the mean absorbance of control wells containing sera from naïve unimmunized mice.

### 2.11. Indirect Serum Neutralization Assay

The serum neutralization assay was adopted from established protocols with slight modifications [28,29]. Briefly, each well of 96-well cell culture plates was filled with 1 × 10^4^ TUBO cells, followed by overnight incubation at 37 °C with 5% CO_2_ to allow cells attachment. Then, sera samples from vaccinated mice in media (%*v*/*v*) were added to the wells containing TUBO cells suspension at 1:1 ratio and incubated at 37° C for 24 h with 5% O_2_ and 5% CO_2_. Sera samples from mice immunized with normal saline were used as a positive control. Cells suspension without sera was serve as the negative control. Then 20 µL of MTT solution was added to each well and incubated for 4 h. The media from each well was carefully removed and replaced with 100 µL DMSO solution to dissolve the formed purple formazan crystals. The recorded absorbance (at 570 nm wavelength) of formazan was proportional to the number of viable cells using spectrophotometry (Infinite M200 PRO, Tecan Trading AG, Männedorf, Switzerland). The experiment was performed in triplicate. The absorbance values in dose-response curves (percentage of serum neutralization vs. concentration) were generated using linear regression interpolation analysis to obtain a minimum concentration of sera that can neutralize the TUBO cell population. The histogram for cell viability was constructed by using GraphPad Prism Software 6.0. Human embryonic kidney 293 (HEK) cells were used to compare serum specificity of immunized mice against TUBO cells.

### 2.12. Establishment of BALB/c Mice Model of TUBO Breast Tumor

The breast tumor engraftment was achieved by injecting 5 × 10^5^ TUBO cells orthotopically into the left lower fourth inguinal mammary fat pads of BALB/c mice under sterile conditions. Mice were observed twice a day (morning and afternoon) for clinical abnormalities, the onset of clinical or toxicological symptoms. After the tumor mass became palpable at day 14, tumor size was measured using a digital caliper, and the volumes were calculated using the formula: V = 1/2 (width^2^ × length). The presence of the tumor was confirmed by radiography diagnostic X-ray (orange 1060HF). The image was generated at 58 kV with a radiation intensity of 0.5 mAs. The tumor tissues were collected from the mice using a 25 G needle via the fine-needle aspiration technique. The samples were stained with Romanowsky stains and subjected for cytopathology examination by using Olympus BX40-B microscope at 40× magnification.

### 2.13. Prophylactic TUBO Challenge Experiment

In the prophylactic experiment, BALB/c mice (6 per group) were subcutaneously injected with 30 µg immunogens (KLH–GP2–P4 and KLH–GP2) in a total volume of 100 µL normal saline on day 0 and subsequently received two booster doses at 14-day intervals (days 14 and 28). The negative control group was administered with normal saline only. Two weeks prior to the final immunization, TUBO cells (5 × 10^5^ TUBO cells in 100 µL media) were injected orthotopically into the left lower fourth inguinal mammary fat pad of the immunized mice. Tumor growth was measured using a digital caliper, and the tumor volumes were calculated by using the following formula:

Tumor volume (mm^3^) = 1/2 (Width^2^ × Length).

The median survival time (MST) was calculated based on the time taken when 50% of the mice died after the vaccination began. Time-to-endpoint (TTE), which is the time taken for the tumor volume to reach 1500 mm^3^, was determined based on the equation of exponential regression of the tumor growth volume. The tumor growth delayed (TGD) percentage was obtained using the following formula:

$$\mathrm{TGD}\;\left(\%\right):\frac{\mathrm{TTE}\;\mathrm{of}\;\mathrm{vaccinated}\;\mathrm{mice}-\mathrm{TTE}\;\mathrm{of}\;\mathrm{unvaccinated}\;\mathrm{mice}}{\mathrm{TTE}\;\mathrm{of}\;\mathrm{unvaccinated}\;\mathrm{mice}}\times 100\%$$

Additionally, the increased lifespan (ILS) of each mouse was calculated using the following formula:

$$\mathrm{ILS}\;\left(\%\right):\frac{\mathrm{MST}\;\mathrm{of}\;\mathrm{vaccinated}\;\mathrm{mice}-\mathrm{MST}\;\mathrm{of}\;\mathrm{unvaccinated}\;\mathrm{mice}}{\mathrm{MST}\;\mathrm{of}\;\mathrm{unvaccinated}\;\mathrm{mice}}\times 100\%$$

Based on animal ethics consideration, mice were euthanized when they have met the following criteria; body weight loss over 15% of the initial weight, tumor volume exceeding 1500 mm^3^ or failure of mice to feed normally due to sickness.

### 2.14. Therapeutic TUBO Challenge Experiment

In a therapeutic experimental setting, 5 × 10^5^ TUBO cells were injected into the left lower fourth inguinal mammary fat pads of the BALB/c mice (6 per group). When the tumor sizes reached approximately 8 mm in the longest dimension, 14 days after tumor inoculation, the mice were administered (similar working concentration as above) with KLH–GP2–P4 or KLH–GP2 or normal saline three times at a two-week interval via subcutaneous injections mice were monitored on a daily basis, and the dataset relevant to tumor analysis was determined based on the aforementioned calculations in the prophylactic experiment.

### 2.15. Statistical Analysis

One-way ANOVA, Tukey’s test, Dunnett’s multiple comparisons test and Log-rank (Mantel–Cox) test were used to determine the statistical validity and significance between the tested groups. The statistical analysis tests were carried out using the GraphPad Prism Software 7.0. Statistical significance was set as follows: ns *p* > 0.05; * *p* < 0.05; ** *p* < 0.01; *** *p* < 0.001; **** *p* < 0.0001.

## 3. Results

### 3.1. Antibodies and Cytokine Responses to Vaccination with Peptide Vaccine Candidates

The BALB/c mice were immunized three times at 14-day intervals, and the sera and splenocytes were collected after 2 weeks of the last immunization (Figure 2A). The capability of KLH–GP2 and KLH–GP2–P4 in inducing HER2/neu-specific IgG antibodies were detected by ELISA, as shown in Figure 2B. Mice immunized with both vaccine candidates elicited significant IgG productions compared to the negative control group. The IgG antibody titers elicited by mice immunized with KLH–GP2–P4 were significantly higher (p < 0.05) than KLH–GP2. A panel of cytokines (IFN-γ, IL-2 and IL-4) secretions from the splenocytes of immunized mice were quantified and compared to the control group (Figure 2C). In general, the expression of IFN-γ and IL-2, which corresponded to Th1 polarization, was significantly higher in splenocytes of mice immunized with both peptide vaccines compared to the control group. The IFN-γ was produced at comparable levels between KLH–GP2–P4 and KLH–GP2, while IL-2 secretion was statistically higher in KLH–GP2–P4 vaccinated group than the KLH–GP2. However, IL-4, which is predominantly produced by Th2 cells, was secreted in significant quantities only in splenocytes from mice immunized with KLH–GP2–P4. Collectively, these immunogenicity data demonstrated that the addition of the B-cell epitope in the peptide vaccine constructs has a profound effect on inducing both cell-mediated and humoral immune responses.

### 3.2. Indirect Serum Neutralization Assay

**Figure 3:**
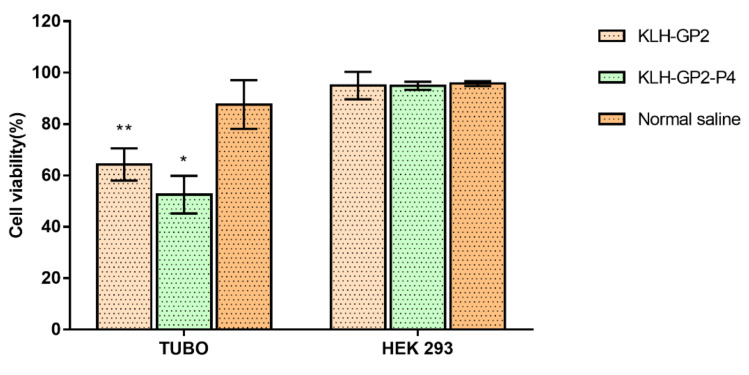
The cell viability of TUBO and HEK293 cells following treatment with sera from the immunized mice. All values are expressed as mean (*n* = 3) ± SEM. The comparison between treated (normal saline, KLH–GP2 and KLH–GP2–P4) and untreated cells (vehicle control group) was evaluated using one-way ANOVA followed by Dunnett’s multiple comparison test (* *p* < 0.05 and ** *p* < 0.01).

The neutralization activity of the sera from immunized mice was tested against TUBO and HEK293 cells (Figure 3). The viability of the TUBO cells was greatly reduced to approximately 52.58 ± 5.19% and 64.28 ± 6.28% when treated with sera from mice immunized with KLH–GP2–P4 and KLH–GP2, respectively, compared to the untreated cells. The TUBO cells viability remained above 80% upon incubation with normal saline as the positive control. It is important to note that the neutralizing activity of KLH–GP2–P4 towards TUBO cells is statistically greater than KLH–GP2 group. The neutralizing activity of the sera from mice immunized with either KLH–GP2–P4 and KLH–GP2 was highly antigen-specific as the cytotoxicity effect was not observed in cell lines that were lacking HER2/neu expression, as tested in HEK293 cells (cells viability > 90%).

### 3.3. Reactogenicity and Safety Evaluations of Peptide-Based Vaccine Candidates in Mice

**Figure 4:**
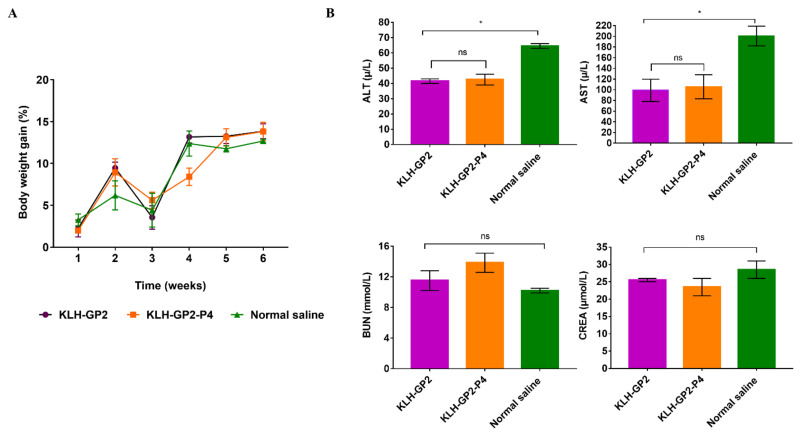
(**A**) Average percentage of weekly measurements of mice body weight during the immunization schedule. (**B**) Serum biochemical parameters (ALT, AST, BUN and CREA) of immunized BALB/c mice. Data presented as mean ± SEM. (* *p* < 0.05, and ns: *p* > 0.05) denote significant different as compared to control group.

No death or behavioral changes were observed in all groups of immunized mice. Our observation also revealed that there were no apparent local reactions, such as erythema and edema at the injection site following and throughout the immunization period. The average percentage of body weight of all the immunized groups of mice increased at the end of the immunization schedule (Figure 4A). Data on the biochemical analysis of serum from immunized mice were presented in Figure 4B. Any interruptions to hepatic function could be detected by elevations in the level of alanine aminotransferase (ALT) and aspartate aminotransferase (AST). The levels of ALT and AST in serum from immunized mice were significantly lower (*p* < 0.05) than the negative control group and within the normal range (ALT: 28–132 U/L and AST: 59–247 U/L) for healthy liver [30], indicating that the vaccine candidates did not induce any damaging effects on the liver cells. The blood urea nitrogen (BUN) and creatine (CREA) levels of the serum samples from immunized mice were comparable to the negative control, which demonstrated that the tested vaccines were not toxic to the kidney.

**Figure 5:**
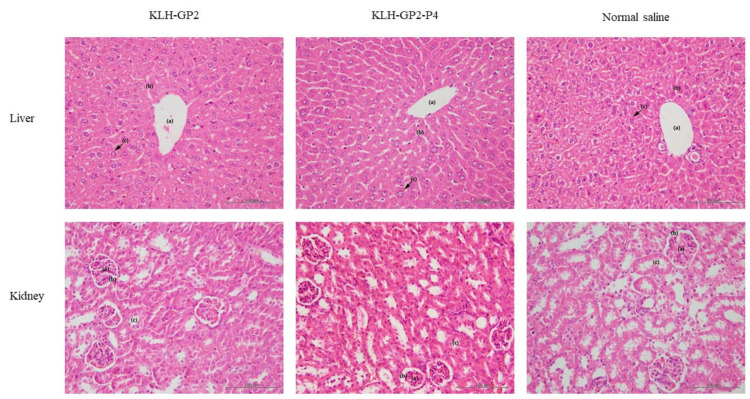
Photomicrographs of liver and kidney sections (H & E stains) from immunized mice. Liver: (**a**) central vein, (**b**) sinusoid and (**c**) hepatocytes. Kidney: (**a**) renal corpuscle, (**b**) capsular space and (**c**) convoluted tubule. Scale bar: 100 μm.

Histopathological assessments of the liver and kidney of the immunized mice are illustrated in Figure 5. The photomicrographs of liver and kidney sections revealed that no abnormalities were detected, thus indicating the safety of vaccine candidates.

### 3.4. Establishment and Validation of TUBO-Derived Mouse Model of HER2/neu Positive Breast Cancer

HER2/neu-enriched tumor was established in BALB/c mice by orthotopic injection of an optimized number of TUBO cells—5 × 10^5^ cells into the left lower fourth inguinal mammary fat. On day 14, after the establishment of TUBO cells, the developed tumor was examined via palpation technique, and the volume was measured using a digital caliper (Supplementary Figure S1). The mean volume of the established tumor was approximately 250 mm^3^. The tumor’s presence was confirmed by X-ray imaging and cytopathology assessment (Supplementary Figures S2 and S3). There were significant radiopaque lesions observed in the mammary region, which indicated tumor formation. The tumor growth is further shown by a cytopathology examination that revealed the irregular presence of monomorphic populations of basophilic cytoplasm and multiple nuclei with hyperchromasia.

### 3.5. Antitumor Efficacy of Prophylactic and Therapeutic Peptide Vaccination

**Figure 6:**
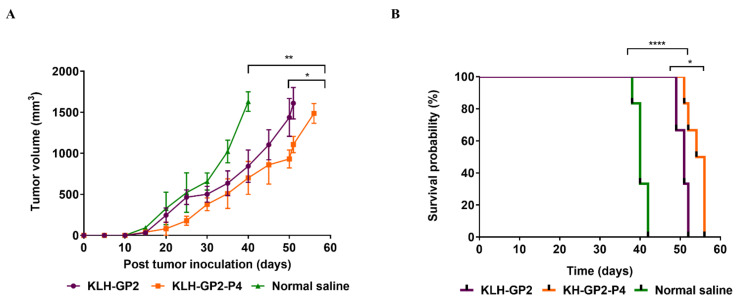
Preventive effects of peptides vaccination against a TUBO tumor in BALB/c mice model. The tumor-bearing mice (*n* = 6) were pre-vaccinated with either KLH–GP2–P4 and KLH–GP2. Mice were examined for (**A**) tumour growth and (**B**) variations in survival. The data are presented as the mean ± SEM. **** *p* < 0.0001, ** *p* < 0.01 and * *p* < 0.05, indicates significant difference as compared to normal saline group.

The efficacy of immune responses produced by KLH–GP2–P4 and KLH–GP2 was compared in a challenge experiment with TUBO cells. The immunized BALB/c mice were challenged with 5 × 10^5^ TUBO cells on day 14 after the final immunization. Tumor development and overall survival of the mice were monitored. As shown in Figure 6A, both KLH–GP2–P4 and KLH–GP2 significantly inhibited tumor growth in comparison to the unvaccinated mice (*p* < 0.01). Notably, KLH–GP2–P4 exhibited greater tumor inhibition than KLH–GP2 (*p* < 0.05). Pre-vaccination with KLH–GP2–P4 and KLH–GP2 also significantly contributed to prolonging the overall survival of tumor-bearing mice compared to the negative control group (*p* < 0.0001), as shown in Figure 6B. The life prolongation indices (MST, TTE, TGD (%) and ILS (%) were generally enhanced in mice vaccinated with KLH–GP2–P4 as opposed to the KLH–GP2-vaccinated group (Table 1).

**Figure 7:**
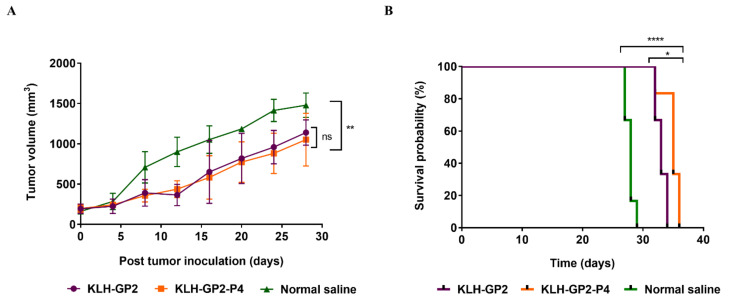
Therapeutic effects of tested vaccine candidates against the TUBO tumor in the BALB/c mice model. The mice (*n* = 6) received three doses of vaccines in two weeks intervals upon tumor establishment. (**A**) Changes in tumor growth and (**B**) the survival time frame of mice. The data indicate the mean ± SEM. **** *p* < 0.0001, ** *p* < 0.01 and * *p* < 0.05 denote significant difference as compared to normal saline group.

The ability of the constructed peptide vaccine candidates in exerting therapeutic effect was evaluated in tumor-bearing mice. Mice were vaccinated on day 14 after the tumor inoculation and establishment. Comparable to prophylactic models, both KLH–GP2–P4 and KLH–GP2 exhibited therapeutic activity by significantly inhibiting the tumor growth in the tumored mice (*p* < 0.01) in comparison to the untreated tumored mice group (Figure 7A). As shown in Figure 7B, the survival rate of the tumor-bearing mice was significantly prolonged when treated with both vaccine candidates compared to the untreated group (*p* < 0.0001). It is important to note that vaccination with KLH–GP2–P4 significantly improved the survival rates of the tumor-bearing mice in comparison to KLH–GP2 (*p* < 0.05). This result was further supported by the data obtained from MST, TTE, TGD (%) and ILS (%) analysis, which demonstrated that vaccination with KLH–GP2–P4 had generated a better therapeutic modality endpoint than KLH–GP2 (Table 2).

## 4. Discussion

Peptide-based vaccines targeting HER/neu have emerged as an attractive modality in breast cancer immunotherapy [31]. However, their clinical application is limited with poor efficacy in the clinical setting. The conventional approach in designing cancer vaccines is primarily aimed at inducing T-cell responses. Nevertheless, vaccine activation of T-cells alone was insufficient, which necessitates the use of a multi-epitope peptide vaccination strategy. The induction of humoral immune response may be biologically significant for antitumor immunity development based on the clinical success of passive immunization with anti-Her2/neu antibodies, as demonstrated by trastuzumab [32].

Therefore, in this study, we have presented an innovative strategy for developing multi-epitope peptide-based HER2/neu for breast cancer by incorporating CTL and B-cell peptide epitopes into a single polypeptide that was linked to KLH as a source of Th epitopes and immunostimulant. The multi-epitope peptide-based vaccine construct was denoted as KLH–GP2–P4 (Figure 1). In our design, a spacer, SGSG, was added between the different epitopes to allow optimal antigen presentation to immune cells [33]. Emphasis was given to elucidating the role of B-cell epitope in harnessing the antitumor immune response by comparing the immunogenicity of KLH–GP2–P4 with its similar counterpart but without the addition of the B-cell epitope (KLH–GP2).

Initially, the immunogenicity affects the constructed vaccine candidates; KLH–GP2–P4 and KLH–GP2 were evaluated in BALB/c mice. As expected, the peptide vaccine possessing the B-cell epitope induced a higher HER2/neu-specific IgG antibody titers than the vaccine construct without the B-cell epitope. Surprisingly, KLH–GP2 was able to elicit a statistically significant antibody response compared to the negative control group. This may be due to the cross-reactive antibodies of KLH to a shared epitope from antigenic determinants of HER2/neu, as observed in other studies that utilized KLH as a carrier protein [34,35]. Alternatively, the IgG antibody response can be evaluated against P4 as a representative of B-cell epitope to obtain an optimal level of more specific antibody productions.

Th cells and cytotoxic T-cells both played distinct roles in inducing specific antitumor immunity [36]. Antitumor vaccinations must therefore induce efficient activation of Th cells and CTL responses. Our cytokines profiling data demonstrated that both peptide vaccine candidates induced high secretion of IFN-γ and IL-2, which were recognized as the indicator of the Th1 response signal (Figure 2C). IFN-γ productions are of great importance for antitumor immunity as it mediates the activation of CTL and natural killer cells, which, in turn, led to the elimination of cancer cells [37]. IL-2 signaling contributes greatly to the priming of T-cells response. We believe that the Th cells’ activation was potentiated by the presence of KLH, which likely led to the enhanced production of IFN-γ and IL-2. Our findings are in line with those studies that used KLH as an adjuvant in the development of peptide-based vaccines [38]. The Th cell proliferation and CTL response were stimulated in an antigen-specific manner in which the sera from immunized mice were found to be selective in inducing cytotoxic effects only against TUBO cells but not towards normal cells (HEK293). It is worth noting that the neutralization activity exhibited by KLH–GP2–P4 was greater than KLH–GP2 (Figure 3). In contrast to KLH–GP2, splenocytes from mice immunized with KLH–GP2–P4 secreted a significant level of IL-4 (governed by Th2 cells), which play a critical role in mediating and maintaining the humoral immune response. Hence, our data demonstrated that humoral immune response plays a significant role in complementing the T-cell-mediated immunity in exhibiting cytotoxic activity against cancer cells.

The TUBO cell line, which expresses HER2/neu oncoprotein, was applied to establish a HER2-positive mouse breast cancer model. The TUBO breast cancer model of BALB/c has been widely used in vaccine development against HER2-positive breast cancer [39,40]. The rat HER2/neu antigen possesses 96% sequence homology to mouse HER2/neu and 88% homologous to human HER2/neu. Thus, the antitumor efficacy of our vaccine candidates was evaluated in the TUBO tumor BALB/c mice model. In the prophylactic challenge experiment, pre-immunization with KLH–GP2–P4 had resulted in a comparable inhibitory effect towards the tumor growth and prolongation rate of mice survival to KLH–GP2 (Figure 6). However, mice immunized with KLH–GP2–P4 had a better endpoint outcome in terms of TGD and ILS by at least 5% and 15%, respectively, compared to KLH–GP2. For therapeutic activity, both KLH–GP2–P4 and KLH–GP2 were capable of significantly inhibited tumor growth in the tumor-bearing mice. Remarkably, the tumored mice that were treated with KLH–GP2–P4 had an improved survival rate compared to the group that was administered with KLH–GP2 (Figure 7B). The superior effects of KLH–GP2–P4 in prolonging mice survival were further supported by the life prolongation indicator, such as MST, TTE, TGD (%) and ILS (%), which was enhanced in tumored mice immunized with KLH–GP2–P4 than KLH–GP2. In particular, the percentage of TGD for mice treated with KLH–GP2–P4 was increased by 7% than mice administered with KLH–GP2. Collectively, our data demonstrated that the addition of B-cell epitope in the peptide-based HER2/neu vaccine design enhanced the antitumor immunity, which was reflected in the greater protective efficacy of KLH–GP2–P4 than KLH–GP2 in both prophylactic and therapeutic vaccination settings. The presence of a B-cell epitope could induce the proliferation of B-cells to mediate vaccine-specific antibodies in generating an antitumor response against targeted cancer cells by facilitating antibody-dependent cell-mediated cytotoxicity (ADCC) [41]. Concurrent with our immunogenicity data earlier, the immune response elicited by KLH–GP2–P4 that was governed by a balanced Th1/Th2 polarization induced a stronger antitumor immunity than KLH–GP2 that was mainly relying on T-cell-mediated immunity. Based on our findings and previous studies [42,43], a balanced stimulation of Th1 and Th2 cells contributed to the enhanced humoral immune response and strengthened T-cell immunity, which resulted in strong suppression of tumor growth.

When it comes to evaluating a vaccine, safety is always the most crucial factor to consider. Both of the constructed HER2/neu-based peptide vaccine candidates were found to be non-toxic, as indicated by serum analysis, cytotoxicity assay, histopathological examination and close monitoring for adverse-reactions symptoms.

## 5. Conclusions

In conclusion, our findings strongly suggest that stimulation of humoral immune response has a significant contribution in mounting an effective antitumor immunity besides T-cell activation. In contrast to KLH–GP2, sera from mice immunized with vaccine construct that bears an additional B-cell epitope (KLH–GP2–P4) elicited a significantly higher titer of antigen-specific antibodies, which resulted in a greater neutralizing effect against TUBO cancer cells (murine HER2-positive cell line). The neutralization effects of KLH–GP2–P4 were governed by a balanced Th1/Th2 polarization, which was confirmed by the elevation of IFN-γ, IL-2 and IL-4 cytokines at a comparable level. Notably, KLH–GP2–P4 generated superior antitumor efficacy over KLH–GP2 not only in prophylactic vaccination but also therapeutically by reducing the tumor growth and enhanced survival of the BALB/c TUBO mice model. Both vaccine candidates were found to be non-toxic. Therefore, our strategy in designing a multi-epitope peptide vaccine with concomitant stimulation of B- and T-cell responses provides a new paradigm in developing preventive and therapeutically effective cancer vaccines.

## Figures and Tables

**Table 1:** Prophylactic efficacy of different vaccine formulations used against a TUBO breast tumor in the BALB/c mice model.

Vaccine Candidates	MST ^a^	TTE ^b^	TGD (%) ^c^	ILS (%) ^d^
KLH–GP2	50	51 ± 2.12 **	27.5 **	25 **
KLH–GP2–P4	56	56 ± 3.27 **	33 ***	40 ***
Normal saline	40	40 ± 1.51	0	0

^a^ denotes median survival time; ^b^ denotes time to reach end point; ^c^ denotes tumor growth delay; ^d^ denotes increased lifespan. ** *p* < 0.01 and *** *p* < 0.001 denote significant difference as compared to normal saline group.

**Table 2:** Therapeutic efficacy of different vaccine formulations used against a TUBO breast tumor in the BALB/c mice model.

Vaccine Candidate	MST ^a^	TTE ^b^	TGD (%) ^c^	ILS (%) ^d^
KLH–GP2	33	34 ± 1.22 *	17 *	21 *
KLH–GP2–P4	35	36 ± 1.51 *	24 **	25 **
Normal saline	28	29 ± 0.75	0	0

^a^ denotes median survival time; ^b^ denotes time to reach end point; ^c^ denotes tumor growth delay; ^d^ denotes increased lifespan. * *p* < 0.05 and ** *p* < 0.01 denote significant difference as compared to normal saline group.

## Data Availability

The data presented in this study can be made available upon request from the corresponding author.

## Supplementary Materials

The following are available online at https://www.mdpi.com/article/10.3390/cancers13194958/s1, Figure S1: Comparison of inoculation sites at day 0 and day 14, respectively, Figure S2: Right lateral thoracic radiograph of mice, Figure S3: Fine needle aspirate finding.
