# Supplementary material for: HER2/neu-Based Peptide Vaccination-Pulsed with B-Cell Epitope Induced Efficient Prophylactic and Therapeutic Antitumor Activities in TUBO Breast Cancer Mice Model
Source: Cancers (Basel). 2021 Oct 1;13(19):4958. doi: 10.3390/cancers13194958 (PMC8507975; doi:10.3390/cancers13194958)
Supplement: Supplementary file 1 [file cancers-13-04958-s001.zip › cancers-1335840-supplementary.pdf]

Supplementary files

HER2/neu-based peptide vaccination-pulsed with B-cell epitope induced efficient prophylactic and therapeutic anti-tumor activities in TUBO breast cancer mice model

Muhammad Luqman Nordin, Abdin Shakirin Mohamad Norpi, Ng Pei Yuen, Khatijah Yusoff, Nadiah Abu, Kue Peng Lim and Fazren Azmi

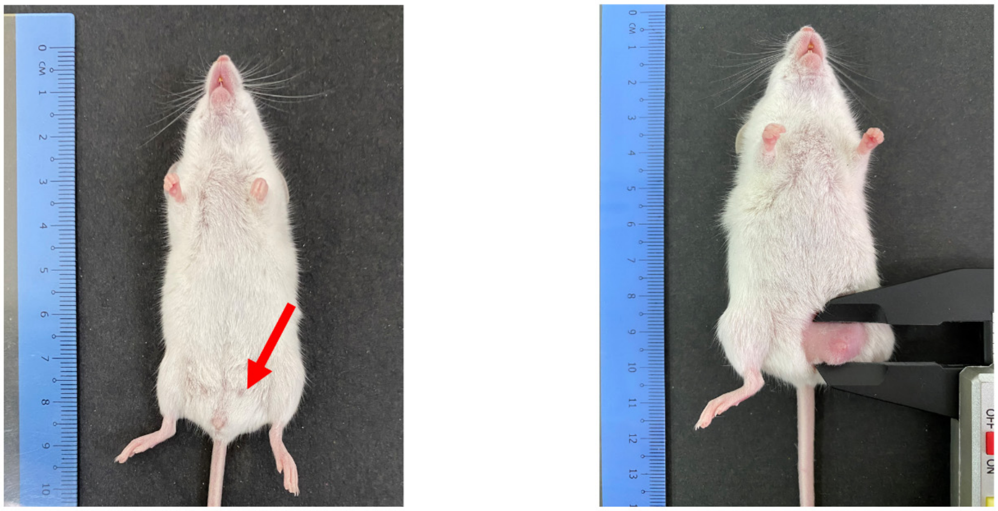

Day 0 Day 14

Figure S1. Comparison of inoculation sites at day 0 and day 14, respectively. Female BALB/c mice were injected with TUBO cell suspension at the left lower fourth inguinal mammary fat pads. The tumor was successfully developed at day 14.

(a) (b)

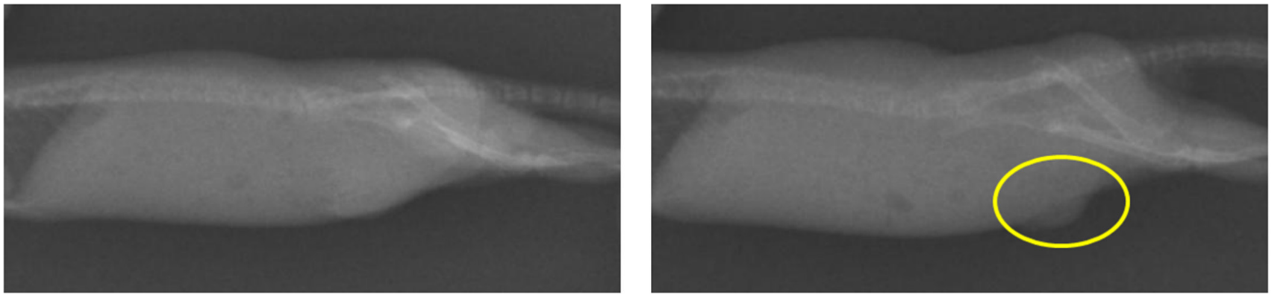

Figure S2. Right lateral thoracic radiograph of mice at day 0 (**a**) and day 14 (**b**) post inoculation of TUBO cells, respectively. Please note that the highlighted image (marked with yellow circle) represented the radiopacity pattern at the mammary region that illustrated the formation of tumor.

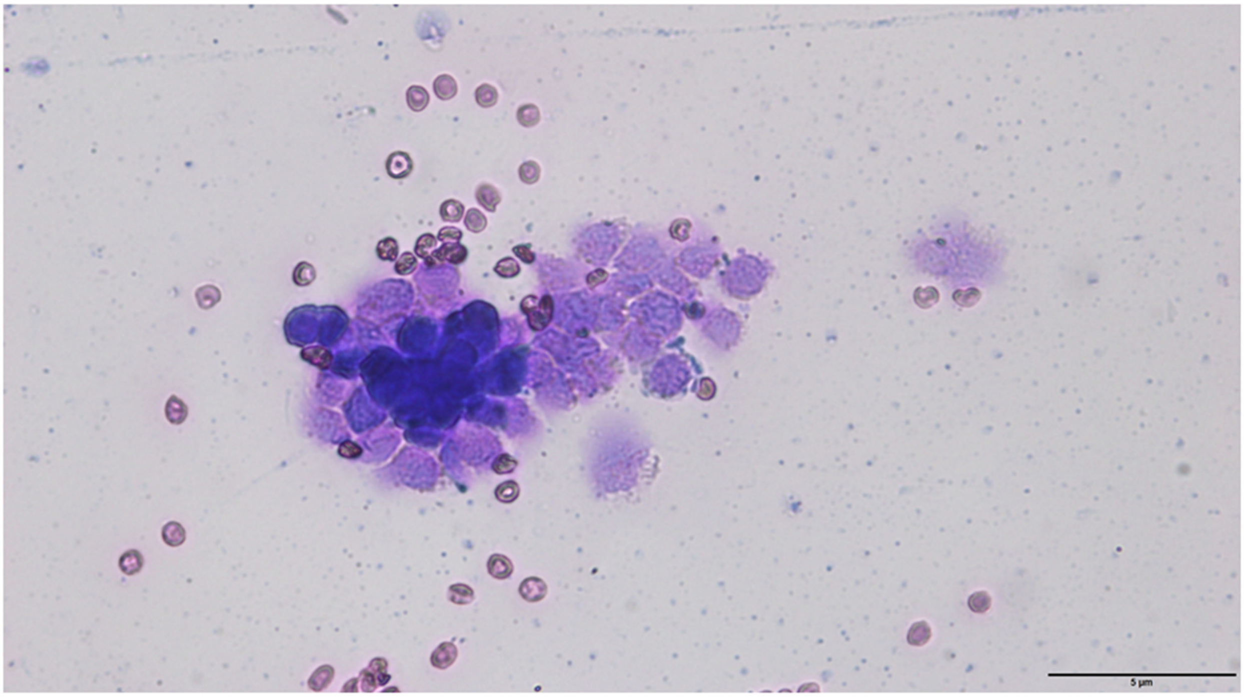

Figure S3. Fine needle aspirate from mice with breast tumor. The monomorphic populations (bluish) with basophilic cytoplasm indicated neoplastic cells that are commonly associated with the presence of benign tumor. There is multiple nucleus with hyperchromasia and gigantism cells features (3x size of erythrocytes) which corresponded to neoplasm characteristics.
